# Circulating Tumor DNA Response and Minimal Residual Disease Assessment in DNA Polymerase Epsilon-Mutated Colorectal Cancer Undergoing Immunotherapy

**DOI:** 10.7759/cureus.43391

**Published:** 2023-08-12

**Authors:** Areeb Lutfi, Maaz K Afghan, Pashtoon M Kasi

**Affiliations:** 1 Oncology, Weill Cornell Medicine, New York, USA

**Keywords:** metastatic colorectal cancer, pole mutation, minimal residual disease assay, cancer immunotherapy, circulating tumor dna (ctdna)

## Abstract

Exonuclease domain mutation (EDM) in polymerase epsilon (*POLE*)-mutated colorectal cancer patients is characterized by specific clinical features and a very high tumor mutation burden (TMB). The therapeutic effectiveness of immune checkpoint inhibitors (ICIs) for the treatment of colorectal cancer in patients with *POLE* mutations is poorly defined. Our case represents a young-onset colon cancer patient who has had a continued response to programmed cell death protein 1 (PD1) blockade alongside clearance of circulating tumor DNA (ctDNA) using a tumor-informed approach. Utilizing ctDNA kinetics to assess minimal residual disease (MRD) in the context of colorectal cancer is a very important topic. Furthermore, utilizing ctDNA kinetics in response to immunotherapy is something that is relevant to all tumor types undergoing immunotherapy. Recently, several landmark articles have proposed this as a promising approach. There is, however, limited information in the literature showing the feasibility of such an approach. Our case report is going to be of value, both from a scientific as well as a clinical standpoint. This is particularly relevant given the rise of colorectal cancers in young individuals.

## Introduction

Immunotherapy in the form of pembrolizumab (PD-1-blockade) has now been approved first-line for patients with mismatch repair deficient/microsatellite instability-high (dMMR/MSI-H) metastatic colorectal cancer (mCRC) based on the results from the Keynote-177 study [1]. PD-1 blockade when combined with a cytotoxic T-lymphocyte-associated protein 4 (CTLA-4) inhibitor too has shown very promising brisk and durable responses [2]. However, dMMR/MSI-H CRC constitutes only 4% to 5% of tumors in the metastatic setting [3]. For the mismatch repair proficient/microsatellite stable (pMMR/MSS) CRC, immunotherapy, unfortunately, does not work [4-5]. The reason why immunotherapy works so well for dMMR/MSI-H CRC versus pMMR/MSS CRC is secondary to the hypermutated nature of mismatch repair-deficient tumors. In addition, this immune sensitivity is given by the continuous generation of new mutations, providing the production of neoantigens activating the immune system over time [6]. In the initial landmark study by Le and colleagues, dMMR/MSI- H CRC had, on average approximately 1782 somatic mutations compared to 73 in pMMR/MSS CRC (p-value = 0.007) [5]. While immunotherapy does not work for pMMR/MSS CRC, one rare exception to this rule is the so-called ultra-hypermutated CRC resulting from a mutation in the exonuclease domain of the catalytic subunit of the DNA polymerase epsilon (*POLE*) [7]. Here, we present a case of a young patient with *POLE*-mutated CRC with near complete ongoing response to immune checkpoint blockade.

## Case presentation

We present a case of a 50-year-old male with a longstanding history of ulcerative colitis who got diagnosed with advanced unresectable colon cancer at the hepatic flexure with extension into the peri-colonic fascia in the setting of hematochezia. The patient's medical history was significant for a 50-pound weight loss in the last five months preceding the diagnosis alongside worsening abdominal pain. On imaging, there were concerns for multiple enlarged abdominal lymph nodes as well as the potential involvement of the second and third part of the duodenum by the hepatic flexure carcinoma. The cancer was considered to be unresectable and potentially metastatic; therefore, systemic therapy was recommended. A biopsy of the hepatic flexure mass came back as adenocarcinoma with squamous differentiation. Given his otherwise young age and good performance status, the patient received triplet chemotherapy with targeted therapy (FOLFOXIRI with bevacizumab). He had an excellent clinical and radiographic response allowing for conversion surgery. Final pathology came back as ypT4bN0 (0 out of 30 lymph nodes), with no evidence of metastases and grade 3 tumor regression was noted. However, there were concerns about the margins being positive on the duodenal side as well as tumor deposits noted with the specimen. The patient underwent a subtotal colectomy given his prior history of inflammatory bowel disease.

**Figure 1:**
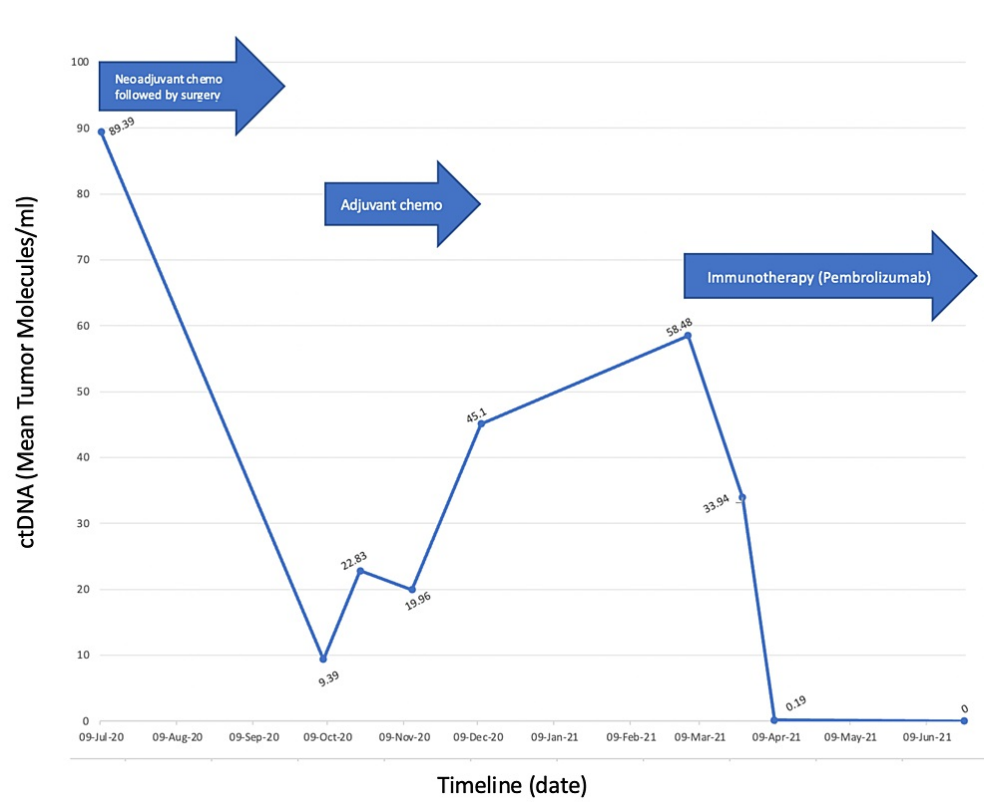
Circulating tumor DNA (ctDNA) kinetics in a patient with POLE-mutant colorectal cancer undergoing therapy using the tumor-informed approach. The patient received FOLFOXIRI and bevacizumab as neoadjuvant chemotherapy and a continued regimen of FOLFOXIRI only as adjuvant chemotherapy.

Postoperatively, the patient received adjuvant therapy consisting of the same regimen of FOLFOXIRI with the omission of the targeted therapy (bevacizumab). While radiographically, there was no evidence of disease before initiation of adjuvant chemotherapy, the patient's tumor-informed minimal residual disease (MRD) circulating tumor DNA (ctDNA) assay using a commercial platform (Signatera^TM^) under the expanded access program was reported positive (Figure 1). A timeline of the chemotherapy the patient received along with the ctDNA levels are reported in Figure 1. Despite the completion of the planned six months of adjuvant systemic chemotherapy, the ctDNA assay continued to remain positive and continued to rise. Follow-up imaging postcompletion of adjuvant therapy now revealed an exophytic soft tissue thickening around the duodenum upstream from the small bowel anastomosis narrowing the lumen and circumferentially encompassing the superior mesenteric vein causing high-grade stenosis. Peritoneal deposits were also visualized in the right hemipelvis adjacent to the colonic anastomosis. All these findings were suspicious of recurrence.

The tumor specimen was sent to commercially available next-generation sequencing (NGS) testing (FoundationONE^TM^) given the concerns of persistently positive ctDNA assay (Figure 1). To our surprise, while the tumor was noted to be microsatellite stable (MSS), it was noted to be ultra-hypermutated with a tumor mutation burden (TMB) of 295 mutations per megabase (Muts/Mb). This is secondary to pathogenic P286R mutation in the polymerase epsilon, catalytic subunit (*POLE*) exonuclease domain. Given these findings, the patient was started on immunotherapy with single-agent pembrolizumab at the approved 400 mg Q6 weekly dosing with rapid clearance of ctDNA (Figure 1) and imaging consistent with a near complete response. On imaging, a significant interval decrease in the size of the mass as well as the implants in the peritoneum was reported. No new lesions were noted.

## Discussion

Immunotherapy in the form of a PD-1 blockade with pembrolizumab has been approved in a tumor-agnostic fashion for any tumor with a TMB ≥10 Muts/Mb as determined by a Food and Drug Administration (FDA)-approved test on June 16, 2020 [8]. While this may be a reasonable approach for a lot of tumors, in the context of CRC, this approval is often criticized since the pMMR/MSS tumors that have a TMB ≥10 Muts/Mb do not derive benefit from immunotherapy [4]. Patients with dMMR/MSI-H CRC generally have a significantly higher TMB, which potentially leads to more expression of neo-antigens and, in turn, response to immune checkpoint blockade. However, one rare exception to this rule, as noted in our case, is the* POLE*-mutated CRC.

POLE-mutant CRC is a very rare finding. The *POLE*-gene encodes the DNA polymerase epsilon; important in correcting replication errors and is most commonly seen in endometrial cancers [9]. A cohort study, including more than 47,000 patients of all cancer types reported *POLE/POLD1* mutations, with the percentage of mutations as high as 14.8% in endometrial cancer [10].

Table 1 summarizes to the best of our knowledge, all reported cases in literature for *POLE-*mutant colorectal cancer treated with immunotherapy. Most common mutations include but are not limited to; P286R, S297F, and V411L [11-12]. As mentioned earlier, our patient's tumor was also noted to have the P286R mutation leading to an extremely high tumor mutation burden. In the context of CRC, these tend to be more right-sided and more often seen in men, as seen in our case (Table 1) [13]*.*

**Table 1:** The table shows the patient's age and sex; tumor location, POLE mutation, immunotherapy received; response to treatment; overall survival, and tumor mutational burden Abbreviations: NED, no evidence of disease; CR, complete response; PR, partial response

Case no.	Age	Sex	Cancer Location	POLE Mutation	Immunotherapy received	Survival Status	Overall Survival (mos)	Tumor Mutational Burden (TMB; mutations/megabase)	Study
1.	50	Male	Hepatic Flexure	P286	Pembrolizumab	Alive (NED)	>24	295	
2.	81	Male	Hepatic Flexure	V411L	Pembrolizumab	Alive (CR)	12	122	Gong et al. [14]
3.	>30	Male	Rectum	P286	Toripalimab	Alive (NED)	12	453	Wen et al. [15]
4.	>70	Male	Ascending Colon	P286	Sintilimab	Alive (NED)	18	255	
5.	>20	Male	Sigmoid Colon	P286	Sintilimab	Alive (NED)	7	320	
6.	>30	Male	Transverse Colon	P286	Toripalimab	Alive (NED)	14	307	
7.	28	Female	Transverse Colon	P286	Ipilimumab + nivolumab	Alive (PR)	-	198	Keenan et al. [12]
8.	37	Male	Ascending Colon	P286	Pembrolizumab	Alive (Significant Response)	>24	168	
9.	44	Male	Rectum	V411L	Pembrolizumab	Alive (CR)	>28	200	Silberman et al. [7]
10.	20	Male	Sigmoid Colon	P286	Pembrolizumab	PD	-	-	Wang et al. [16]
11.	34	Male	Cecum	P286	Pembrolizumab	Alive (SD)	>7	-	
12.	82	Male	Ascending Colon	V411L	Pembrolizumab	Alive (CR)	>12	-	
13.	24	Male	Descending Colon	P286	Pembrolizumab	Alive (CR)	>48	126	Durando et al. [17]
14.	55	Male	Cecum	P436	Pembrolizumab	Alive (CR)	>24	>150	Bikhchandani et al. [18]
15.	16	Male	Transverse and Descending Colon	Ser297Cys* Lynch Syndrome	Nivolumab + anti LGA3	Alive (CR)	>18	530	Berrino et al. [19]
16.	34	Male	Transverse Colon	P286	Terriprizumab and bevacizumab	Alive (PR)	>18	120	Xiang et al. [20]

Our case is unique from a therapeutic standpoint since while aggressive systemic chemotherapy and surgery rendered the patient with no evidence of disease, molecularly, from an MRD or ctDNA standpoint, the patient was still positive (not cured) [21-22]. Not surprisingly, radiographic recurrence was noted a month post-completion of adjuvant therapy. What is also of value to note is the ctDNA kinetics in predicting response to immune checkpoint blockade [23]. ctDNA started going down within eight days of administration of the first dose of immunotherapy (Figure 1). It is often underestimated how quickly immunotherapy responses can be seen at the molecular level. Furthermore, the ultra-hypermutated nature of the neoplasm leading to the significantly high TMB is indeed a predictive marker of response to immunotherapy for this subset of pMMR/MSS CRC. While rare, it is important especially in the context of individuals with young onset colorectal cancer to not only have panel-based testing but also to have the TMB checked as well [13]. Responses here to immunotherapy are brisk, deep, and durable - similar to dMMR/MSI-H CRC [7,12,24-25].

## Conclusions

There is a need to identify subsets of CRC that would benefit from an immunotherapy approach besides those with dMMR/MSI-H tumors. Patients with pMMR/MSS CRC that have the POLE or other polymerase mutations leading to an ultra-hypermutated phenotype are another subset that can benefit from immunotherapy. With the rise of colorectal cancers in the young, which are often diagnosed as advanced/metastatic, panel-based NGS testing, including TMB assessment should be the norm.
